# The electronic and optical properties of Cs_2_BX_6_ (B = Zr, Hf) perovskites with first-principle method

**DOI:** 10.1371/journal.pone.0292399

**Published:** 2023-12-22

**Authors:** Yang-Yang Zhao, Si-Yuan Sheng

**Affiliations:** 1 Department of basic education, Criminal Investigation Police University of China, Shenyang, Liaoning, China; 2 Department of Physics, Shenyang University of Chemical Technology, Shenyang, Liaoning, China; Nazarbayev University, KAZAKHSTAN

## Abstract

The electronic structures and absorption properties of Cs_2_BX_6_ halide compounds are investigated with first principle calculation and exchange correlation functional of GGA-PBE. Pressure and halogen ion doping are employed to regulate band gap. All materials suffer transition from indirect to direct band gap semiconductors but with different phase transition pressure. Structural and band structure calculating results show that the value of phase transition pressure is mainly determined by the volume of octahedron. When the volume of vacancy octahedron is much less than B-ion octahedron, the lowest band point of B-d orbitals transforms to Γ point, then the indirect semiconductors transform into direct band gap semiconductors. Calculating results of optical absorption implied that the systems have obvious blue shift, which result in the optical properties reduced. Based on suitable band gap and higher absorption coefficient, Cs_2_ZrI_4_Br_2_ can be an ideal candidate for perovskites solar cells.

## Introduction

The lead-free halide perovskites have been widely studied for the last few years [1–4]. Compared with the traditional semiconductors, halide perovskites have attracted great attention owing to their high absorption coefficient, defect-tolerance, long diffusion length, appropriate band-gap [5–7]. They are widely used in photovoltaic solar cells, photoelectric detector, laser device, etc [8, 9]. However, many of these halide perovskites suffer two major issues of environmental stability [3, 10, 11] and low power conversion efficiency(PCE) [12]. These troubles currently preclude their widespread in commercial applications. Therefore, there is a strong desire to find environmentally stable and high efficiency perovskite materials. In order to resolve these problems, many groups have done a lot of theoretical and experimental researches.

The basic crystal structure of photovoltaics perovskite is ABX_3_. In inorganic perovskite A usually for Cs^+^, X represents halide ion, and B is divalent metal cation, whose volume is smaller than A. In the past few years, significant theoretical efforts and experiments have been made to understand properties of energy band and photovoltaic of these materials. Obviously, the most commonly used lead-free perovskite materials is set B with Sn^2+^ and Ge^2+^ [13–17]. But both of them suffer from much serious instability issues [18], and the lower PCE is also a troublesome problem [19]. Then the family of vacancy ordered double perovskites A_2_BX_6_ have been widely discussed as well [20, 21]. Results show that energy and optical properties are mostly determined by [BX_6_] octahedral, doping and pressure also have extraordinary effects on characteristics of lattice parameter, energy gap, optical absorption coefficient and so on. In 2017, group of Yao Cai completed the calculation of chemical trends and structural stability of A_2_BX_6_ (A = K, Rb, Cs; B = Pd, Pt, Sn, Te; X = Cl, Br, I) [22]. Their findings provide guidelines for the design of halide A_2_BX_6_ compounds. Muhammad Faizan et al. found Rb_2_PdBr_6_ and Cs_2_PtI_6_ were suitable for single-junction photovoltaic applications [23]. Some researchers have also shown that the band gap of materials could be regulated by doping alloying elements on B-site [24].

Judging from material stability, we prefer to choose Cs as A cation. In general, most theoretical and experimental results have indicated that Cs_2_TiX_6_ is an ideal photoelectric conversion material. Titanium, zirconium and hafnium belong to the same group, so they are some alike in nature stability and structural characters. In this context, through the first-principle calculation based on density functional theory (DFT), the energy and optical properties of Cs_2_HfI_6−*x*_X_*x*_ and Cs_2_ZrI_6−*x*_X_*x*_ with the influence of pressure are studied detailed. Firstly, the structural optimization and stability of the compound was discussed. Secondly, the energy band and density of states was calculated to study the phase transition from indirect to direct semiconductor with the influence of doping and pressure. Then the optical properties were studied. Finally, some conclusions were proposed in the last section of paper.

## Calculating methods

First principle calculation was implemented by using Vienna *ab* *initio* simulation package (abinit) [25]. The exchange correlation function between electrons was described by generalized gradient approximation (GGA) with Perdew-Burker-Ernzerhof (PBE) [26]. The projector augmented wave (PAW) was employed to describe interactions between core and valence electrons [27]. Valence electron configuration-Cl (3*s*2 3*p*5), Br (3*d*10 4*s*2 4*p*5), I (4*d*10 5*s*2 5*p*5), Cs (6*s*1), Zr (4*d*2 5*s*2), Hf (4*f*14 5*d*2 6*s*2). The plane wave energy truncation for 20*hartree*. Structural relaxation using BFGS method [27]. The accuracy of relaxation convergence was set to 1.0 × 10^9^*hartree*/*atom*, and interaction between atoms was less than 1.0 × 10^−5^*hartree*/*Bohr*. When using ab initio molecular dynamics (AIMD) [28] method to verify structure relaxation results, super lattice of 2 × 2 × 2 was adopted and *k*-point grid was Γ(0, 0, 0) only. In this work, the energy fluctuations accuracy converges to 0.25 hartree in 20 *ps*. When calculating the crystal energy and optical properties, 16× 16 × 16 *k* point grid was employed. In order to get more accurate absorption spectrum, the band number was set to triple of valence band.

## Results and discussion

### Structural properties and stability

**Fig 1:**
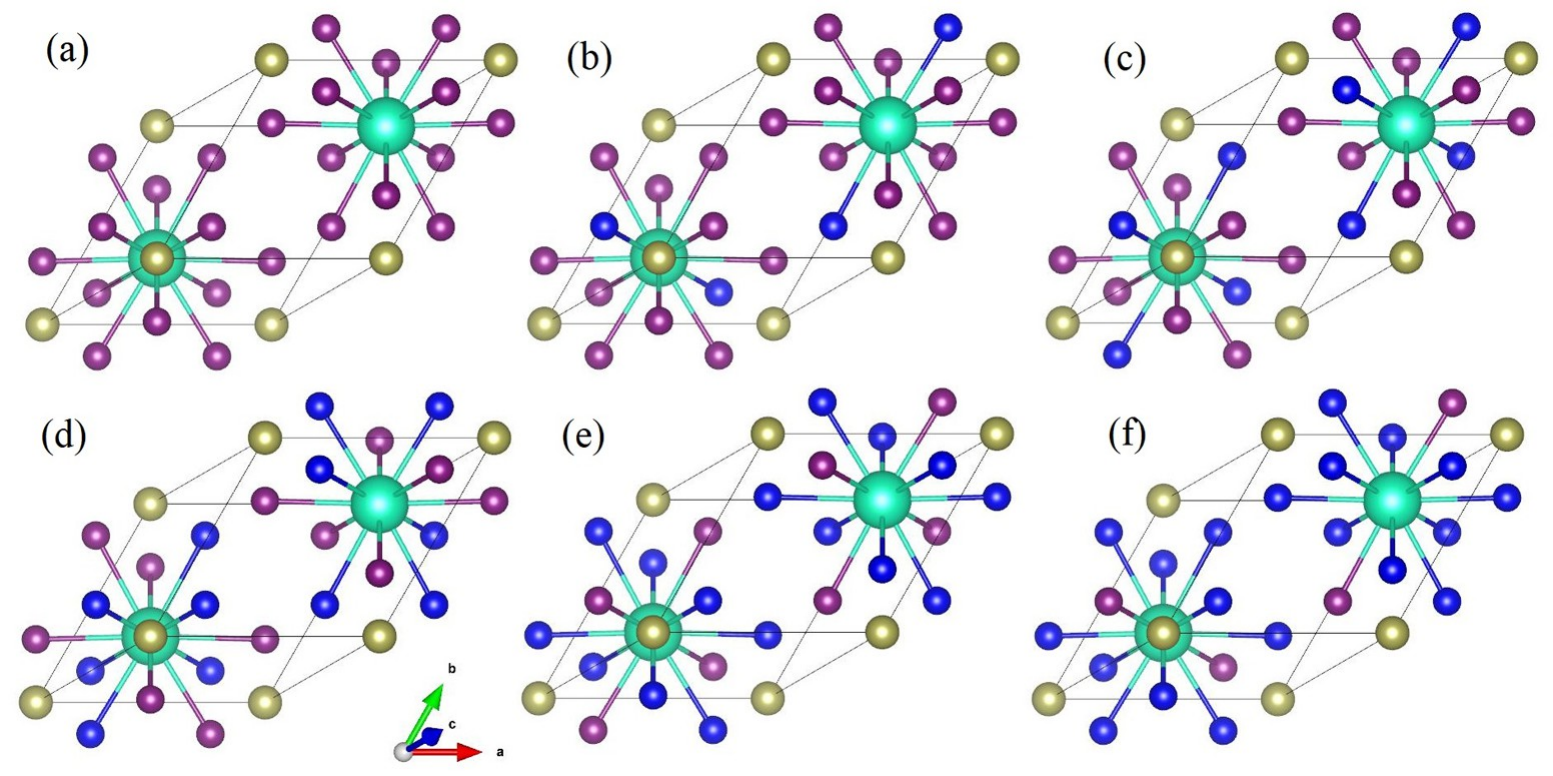
Optimized geometric structures of Cs_2_BX_6_. (a)-(f) shows the optimized structure of materials with doping concentration from *x* = 0 to *x* = 5(balls color: green-Cs, yellow-Hf or Zr, purple-I, and blue means the doping halogen atom).

Fig 1 represents the optimized geometric structures of Cs_2_BX_6_ (B = Hf,Zr) view along the (1 1 0) axis. Fig 1(a)–1(f) shows the structure of materials with doping concentration from *x* = 0 to *x* = 5. We can see that as for Cs_2_BX_6_ perovskites, the structure is a typical configuration of *Fm* − 3*m* cubic crystal. The BX_6_ form stable octahedron configuration, where halogen atoms locate at the vertices of octahedral, and B-site cation locate in the center of the octahedral structure. While A locates in the center of four neighboring octahedral structures. With the doping of halide atom, structures of materials are not standard cubic lattice any more. On the basis of existing results, the photoelectric characteristics of the material is mainly decided by the BX_6_ octahedron configuration [20, 21]. The radius of B and halogen atoms can directly affect the material structure, including lattice distortion, the bond length and bond angle. Besides, external pressure and temperature also have significant influence on lattice structure and optical properties [29–31].

**Fig 2:**
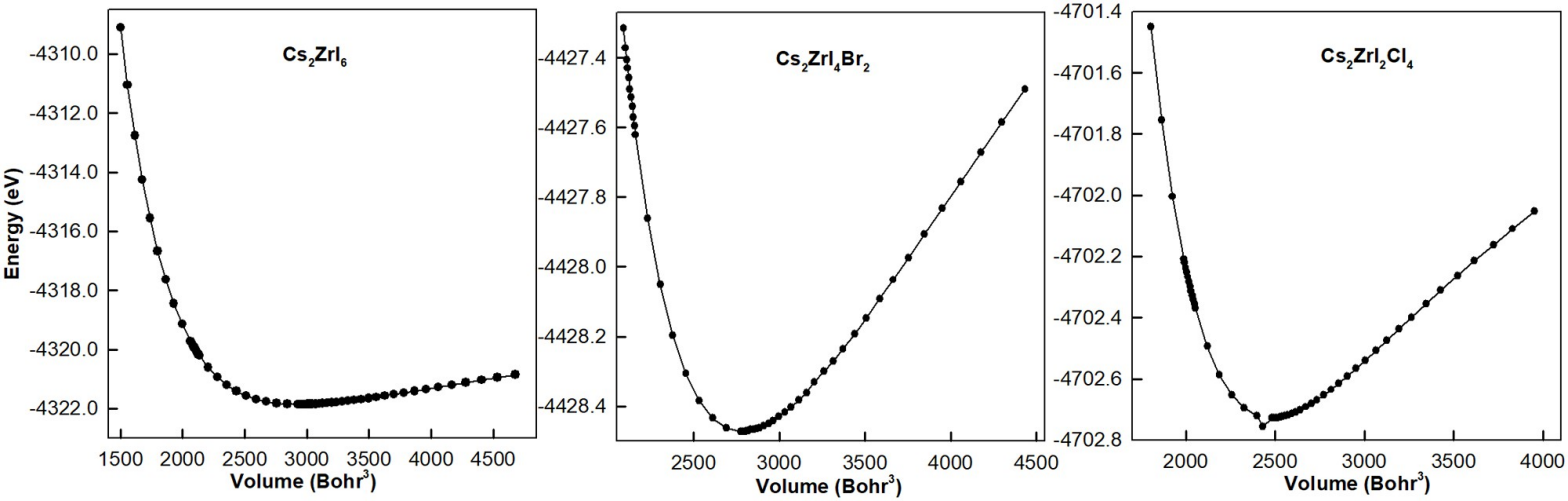
Variation of total energy as the function of volume of Cs_2_ZrI_6_, Cs_2_ZrI_4_Br_2_ and Cs_2_ZrI_2_Cl_4_.

**Fig 3:**
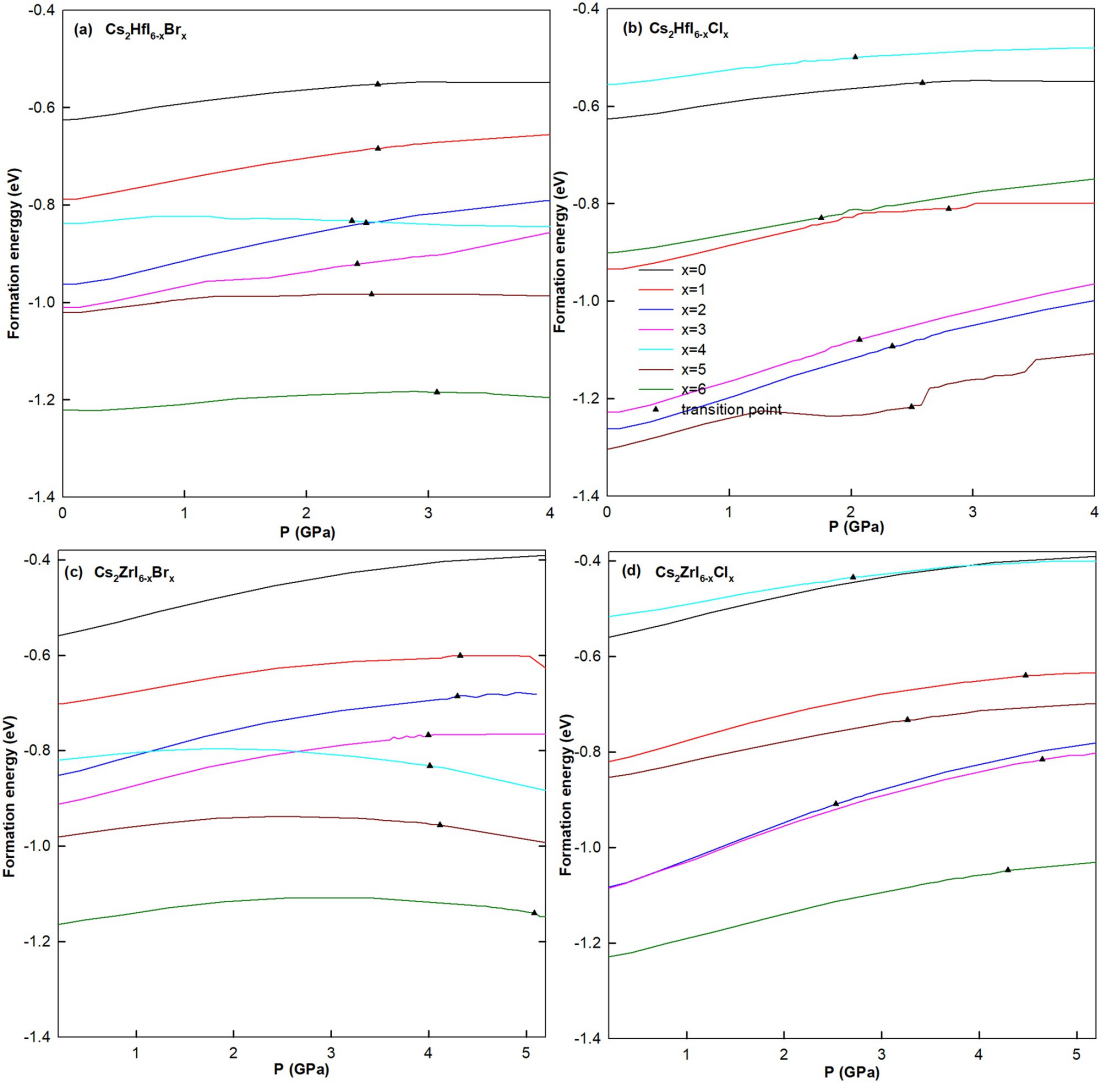
Formation energy of 26 kinds of perovskites with different pressures. Different colours of curves in (a)-(d) stand for different doping concentration *x* of halogen elements. Triangular symbols represent transition point of indirect-direct gap semiconductors.

Then the structural optimized curves of total energy versus volume at P = 0 are shown in Fig 2 to describe the lattice distortion with the influence of doping. The differences between figures indicate that these compounds belong to different crystal systems at equilibrium with P = 0. Compared with the optimized structures shown in Fig 1, we can conclude that in a super cell Cs_2_ZrI_6_ is a cubic structure, Cs_2_ZrI_4_Br_2_ is the tetragonal body centered system, and Cs_2_ZrI_2_Cl_4_ is the orthorhombic body centered structure. The variation of structural optimized curves in this study is similar with that of Cs_2_TiBr_6−*x*_I_*x*_ [30]. But for the compounds studied in this paper, there is little experimental and theoretical evidence to compare with our calculated results, so our data maybe a good reference for future work. The stability of compounds can be described by formation energy as the following equation:

$$\begin{array}{c} E=E_{Cs_{2}BX_{6}}-2E_{CsI}-E_{BI_{4}}, \end{array}$$ (1)

where $E_{Cs_{2}BX_{6}}$, *E*_*CsI*_, and $E_{BI_{4}}$ are the total energies of Cs_2_BX_6_ system, CsI and BI_4_, respectively. With different pressure, all the formation energies of 26 kinds of perovskites with multiple doping concentration shown in Fig 3 are negative, which means that all the materials have excellent structural stability.

### Band structure

**Fig 4:**
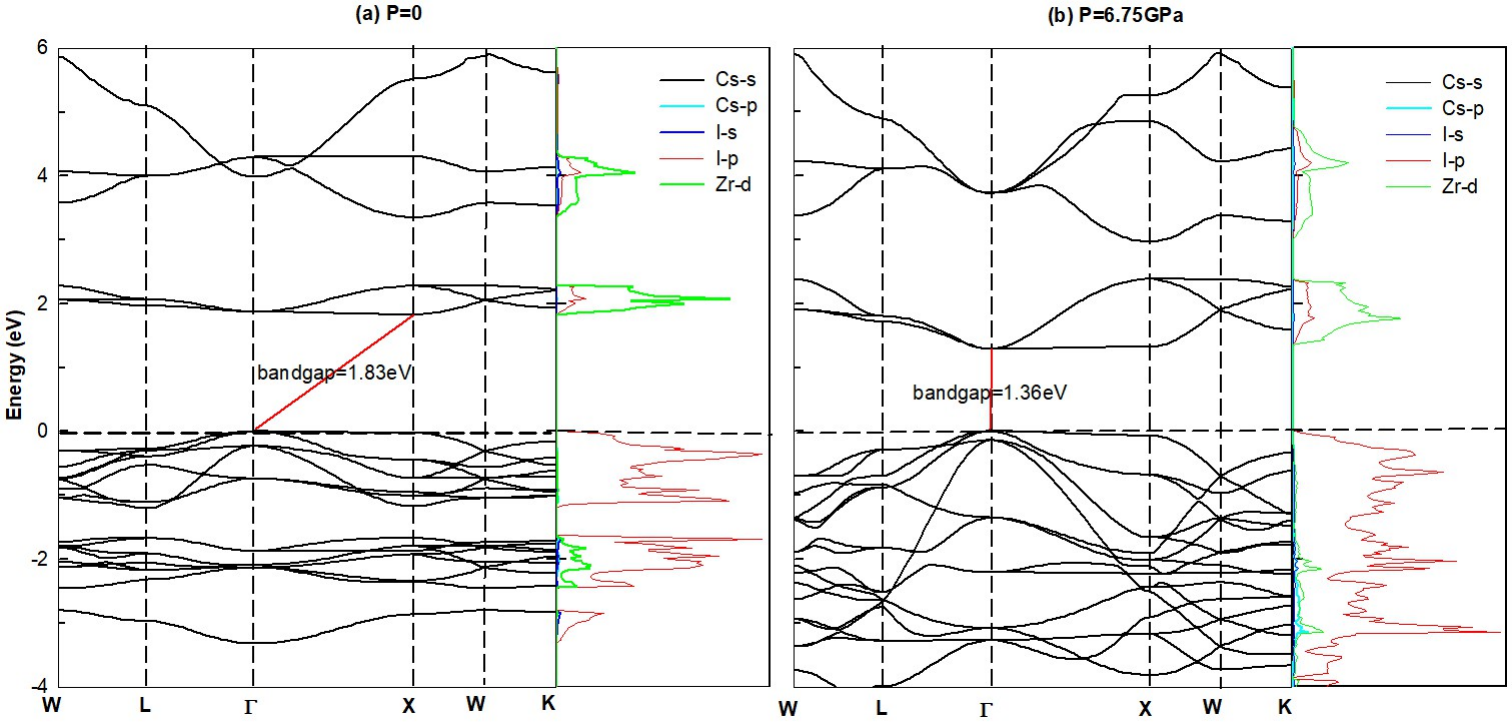
The band structures and density of states of halide perovskites Cs_2_ZrI_6_ with (a) P = 0 and (b) P = 6.75*GPa*. The bandgap is 1.83*eV* and 1.36*eV*, separately. P = 6.75*GPa*: there is a material transition from indirect to direct band gap semiconductor, and the CBM moves from X to Γ-point.

**Fig 5:**
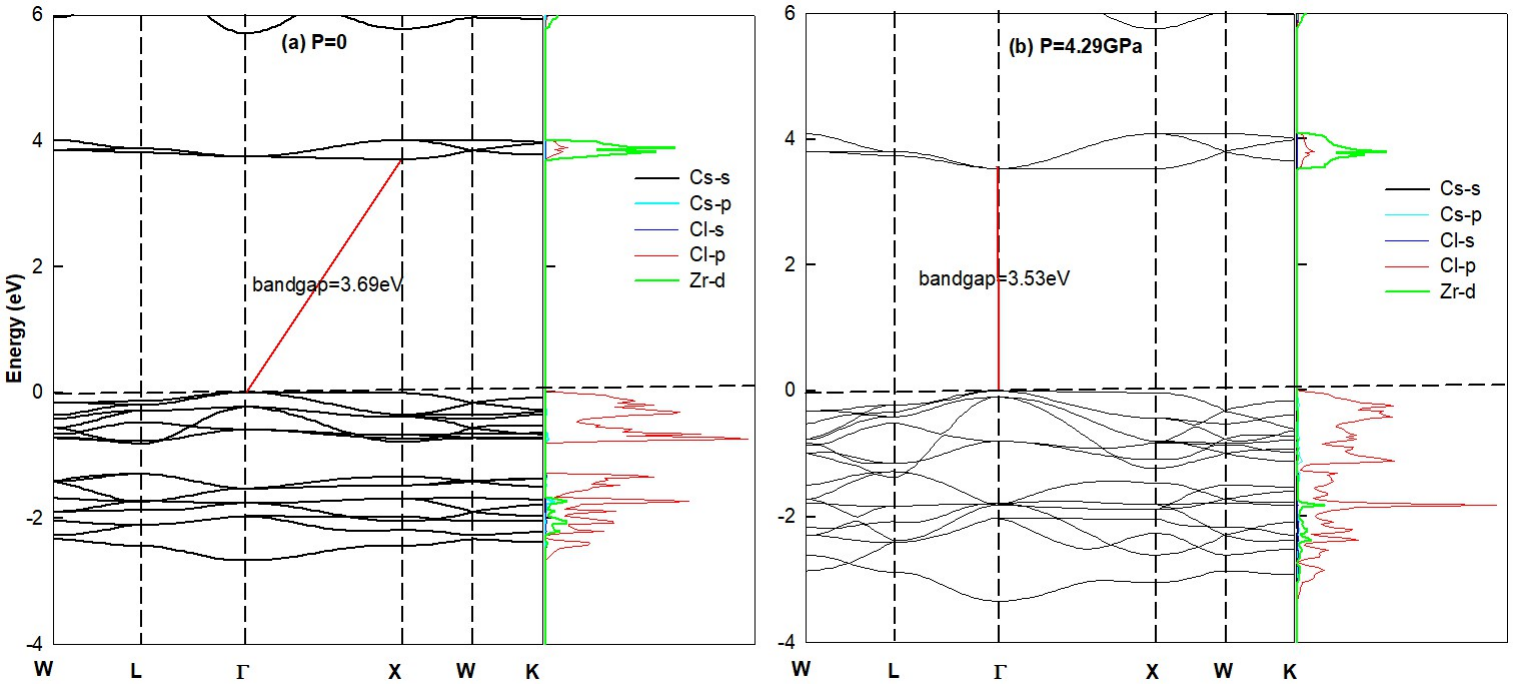
The band structures and density of states of halide perovskites Cs_2_ZrCl_6_ with (a) P = 0 and (b) P = 4.29*GPa*. The bandgap is 3.69 *eV* and 3.53 *eV*, separately. Accompany with material transform from indirect to direct band gap semiconductor, the CBM moves from X to Γ.

To understand the electronic conductivity with different pressure, the band structure and density of states (DOS) were calculated. Take the pure lattice system Cs_2_ZrI_6_ and Cs_2_ZrCl_6_ for example, the results are shown in Figs 4 and 5. In Fig 4, we can see that with P = 0, Cs_2_ZrI_6_ is an indirect bandgap semiconductor with gap value of 1.83*eV*. The top of valence band(VBM) located at Γ(0, 0, 0) is mainly composed by I-4*p* orbital, and the bottom of conduction band(CBM) situated at *X*(0.5, 0.5, 0) is contributed by Zr-3*d* state. While P = 6.75 *GPa*, the material turn into direct bandgap semiconductor with gap value of 1.36*eV*. These results mean that with the increase of pressure, materials suffer from indirect-direct band gap transition. During this process, the VBM always stays at the same position, while the location of CBM moves from X-point to Γ-point, which accompanied with the decrease of energy value. The band structure of Cs_2_ZrCl_6_ in Fig 5 share the same feature with Cs_2_ZrI_6_, but the former has bigger band gap.

The accuracy of density functional theory (DFT) predictions for ground state energies and density distributions can be improved by better hybrid functional for exchange correlation energy. According to Jacob’s ladder of approximate DFT methods, we know that GGA, meta-GGA(mGGA) and HSE06 belong to different level with functional complexity varies greatly. GGA takes density gradient into energy density functional to correct the error introduced by uneven distribution of electron density, and PBE pseudopotential is one of the most useful exchange-correlation functional. The mGGA is a most sophisticated semi-local functional, incorporating important exact conditions with respect to GGA functional. In general, mGGA functional with the kinetic energy density, which enters in the expansion of the angle-averaged exact exchange hole, thus being an important tool in the construction of exchange-correlation approximations. The most popular mGGA functional includes BR89, VSXC, TPSS and so on. The HSE06 functional is an outstanding representative of hybrid-GGA. It takes spin-orbit coupling into consideration so that usually gives bandgaps of heavy metals closer to experimental values than GGA results. This is because that spin-orbital coupling have an important effect on the bandgap by splitting the energy levels around Fermi level.

**Fig 6:**
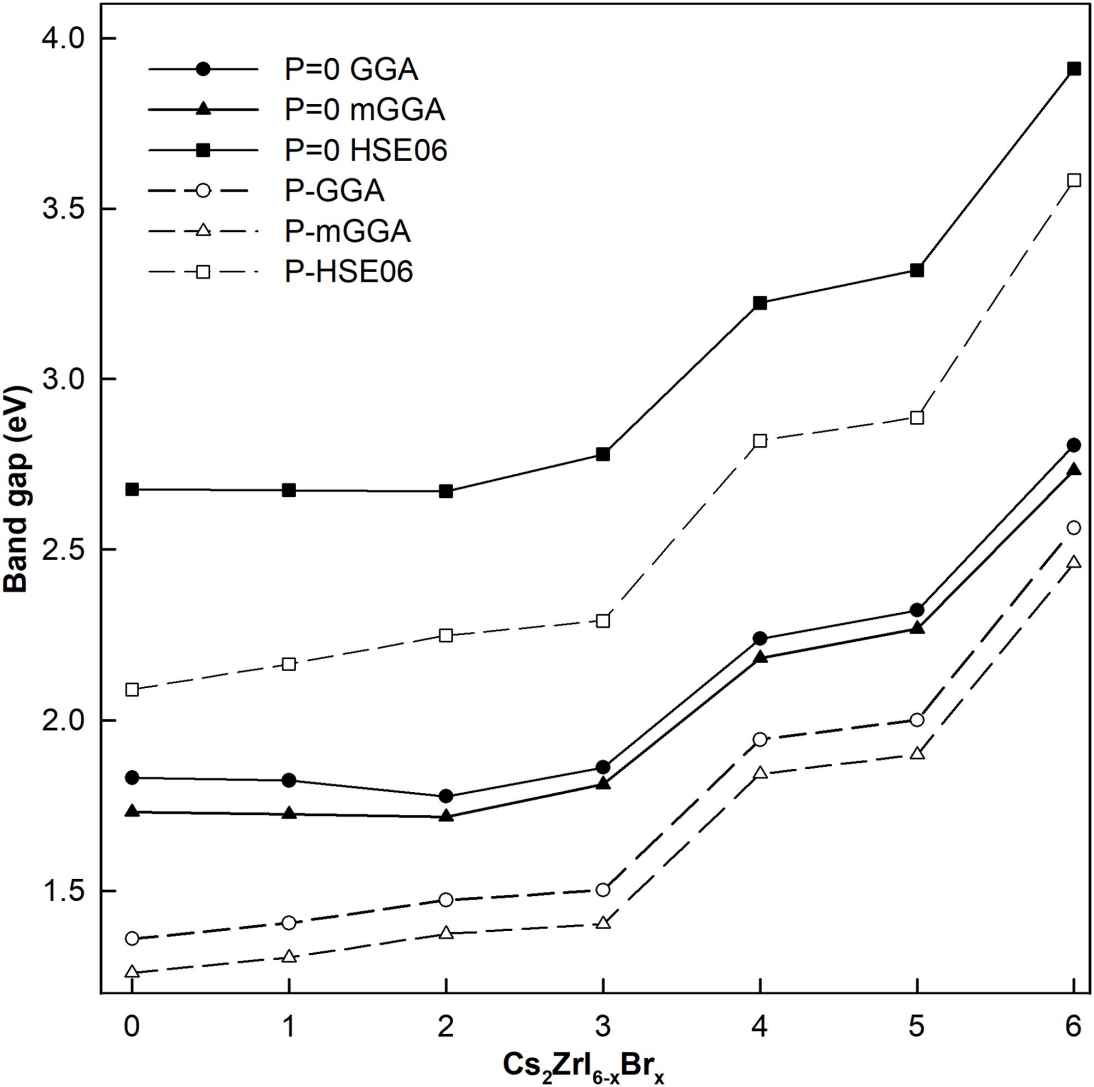
The band gap of Cs_2_ZrI_6−*x*_Br_*x*_ with P = 0(solid line) and phase transition pressure(dashed line) as a function of doping concentration, compared to three kinds of hybrid functional: GGA (circle), mGGA (triangle) and HSE06 (square).

Fig 6 shows the band gaps of Cs_2_ZrI_6−*x*_Br_*x*_ with *P* = 0(solid line) and phase transition pressure(dashed line) as a function of doping concentration, compared to three kinds of hybrid functional: GGA (circle), mGGA (triangle) and HSE06 (square). In this paper, the exchange-correlation energy of HSE06 $E_{XC}^{HSE}$ is described as follows:

$$\begin{array}{c} E_{XC}^{HSE}=1/4E_{x}^{SR}\left(\mu \right)+3/4E_{x}^{PBE,SR}\left(\mu \right)+E_{x}^{PBE,LR}\left(\mu \right)+E_{c}^{PBE} \end{array}$$ (2)

where $E_{x}^{PBE}\left(\mu \right)$ and $E_{c}^{PBE}$ are the exchange and correlation energy functions of PBE, respectively. In general, the gap value of Cs_2_ZrI_6−*x*_Br_*x*_ grows with the increase of Br doping. And the band gaps of materials calculated with GGA and mGGA method are basically consistent, while the results with HSE06 method are highly overvalued. These results agree well with previous theoretical values which indicate that the band gap of halide perovskites obtained by HSE06 is slightly larger than their experimental and other theoretical values [32]. The major reason may be that Zr is a so light element that it is unnecessary to consider electron coupling in band structure calculation. Therefore, in this paper we mainly use GGA hybrid functional method to study the properties of perovskites.

Both the structural change with doping and lattice distortion constructed by pressure can directly affect the electrical and optical properties. From now on, we discuss the competing effects of these two factors. Tables 1 and 2 are the calculated electronic bandgaps of perovskites. For all the 26 halide perovskites, halogen atoms can significantly regulate the material’s band gap. Unfortunately, all the materials are indirect band gap semiconductors. From the point of electronic transport, indirect bandgap semiconductors can also bring electrons from VBM to CBM, while in this process, part of the energy will lost in the form of phonons. This energy dissipation will greatly reduce the life of materials. Therefore, the direct band gap semiconductors are more popular in solar cells application. With the increase of pressure, all materials completed the transformation from indirect bandgap to direct bandgap semiconductor but with different phase transition pressure. Based on experimental data, solar battery materials band gap within the optimal range of 0.9–1.6 eV, and good photoelectric materials ideal band gap between 1.38 eV to 1.78 eV. Combined with our calculation data, materials of Cs_2_ZrI_6_, Cs_2_ZrI_5_Br, Cs_2_ZrI_4_Br_2_, Cs_2_ZrI_3_Br_3_, Cs_2_ZrI_5_Cl, Cs_2_ZrI_4_Cl_2_, Cs_2_ZrI_3_Cl_3_ can be used as solar cells, and Cs_2_ZrI_2_Cl_4_ is a better photoelectric material.

**Table 1:** Indirect bandgap and direct bandgap with pressure of Cs_2_HfI_6−*x*_X_*x*_.

Compound		Indirect gap (eV)	Direct gap (eV)	Pressure (GPa)
Cs_2_HfI_6_		2.2273	1.9507	2.5862
Cs_2_HfI_6−*x*_Br_*x*_	*x* = 1	2.2264	1.9128	2.5868
	*x* = 2	2.2257	1.9492	2.4891
	*x* = 3	2.3410	2.0284	2.4163
	*x* = 4	2.7296	2.4748	2.3721
	*x* = 5	2.8314	2.5540	2.5342
	*x* = 6	3.3137	3.1097	3.0719
Cs_2_HfI_6−*x*_Cl_*x*_	*x* = 1	2.2251	1.8465	2.8014
	*x* = 2	2.2538	1.9814	2.3386
	*x* = 3	2.4180	2.0790	2.0702
	*x* = 4	3.0949	2.8313	2.0349
	*x* = 5	3.1818	2.9243	1.7563
	*x* = 6	4.2470	4.1241	2.4965

With P = 0, both Br and Cl doping the material of Cs_2_HfI_6−*x*_X_*x*_ is indirect band gap semiconductor, and the gap increased with the increase of doping concentration. With different pressure, all materials turn into direct band gap compound, and the pressure shown in table is the phase transition pressure.

**Table 2:** Indirect bandgap and direct bandgap with pressure of Cs_2_ZrI_6−*x*_X_*x*_.

Compound		Indirect gap (eV)	Direct gap (eV)	Pressure (GPa)
Cs_2_ZrI_6_		1.8304	1.3588	6.7519
Cs_2_ZrI_6−*x*_Br_*x*_	*x* = 1	1.8502	1.4053	4.3237
	*x* = 2	1.8122	1.4718	4.2940
	*x* = 3	1.8618	1.5020	3.9963
	*x* = 4	2.2393	1.9428	4.0125
	*x* = 5	2.3217	2.0004	4.1154
	*x* = 6	2.8056	2.5634	5.0840
Cs_2_ZrI_6−*x*_Cl_*x*_	*x* = 1	1.7702	1.3110	4.4788
	*x* = 2	1.8850	1.5545	2.5325
	*x* = 3	1.9110	1.4460	4.6488
	*x* = 4	2.1056	1.7600	2.7081
	*x* = 5	2.6231	2.3035	3.2655
	*x* = 6	3.6893	3.5302	4.2943

With P = 0, both Br and Cl doping the material of Cs_2_ZrI_6−*x*_X_*x*_ is indirect band gap semiconductor. With different pressure, all materials turn into direct band gap compound, and the pressure shown in table is the phase transition pressure.

From Fig 3, we can clearly see that at phase transition point the pressure varies greatly for different materials. For Cs_2_HfI_6−*x*_Br_*x*_ and Cs_2_ZrI_6−*x*_Br_*x*_, the difference of phase transition pressure caused by Br-doping is small. The former is mainly concentrated between 2.5*GPa* and 3*GPa*, while Cs_2_ZrI_6−*x*_Br_*x*_ need more pressure about 4*GPa*. The transition pressure of Cs_2_ZrBr_6_ and Cs_2_ZrI_6_ are almost up to 5*GPa* and 6.75*GPa* separately. As to Cs_2_HfI_6−*x*_Cl_*x*_ and Cs_2_ZrI_6−*x*_Cl_*x*_, the pressure fluctuates greatly near the phase transition point.

Considering the influence of pressure, we find that the trends of phase transition are the same for 26 kinds of perovskites. In general, Cs_2_BX_6_ compounds are considered as vacancy ordered double perovskites with B-periodic deficient of CsBX_3_. Due to the presence of vacancy ordered, double perovskite materials all possess so great elasticity coefficient that the volume of structure will decrease obviously with the increase of pressure. In our opinion, the volume declining rate of vacancy octahedron is higher than that of B-ion octahedron. When the volume of vacancy octahedron is much less than B-ion octahedron, the lowest band point of B-d orbitals transforms to Γ point. At the same time, materials were converted into direct band gap semiconductors. As to different halogen and B-site atoms, elastic coefficients of materials take different values, which lead to octahedral structures have various sensitivity to pressure. This is the reason why materials have different pressure values at the transition point.

### Optical properties

**Fig 7:**
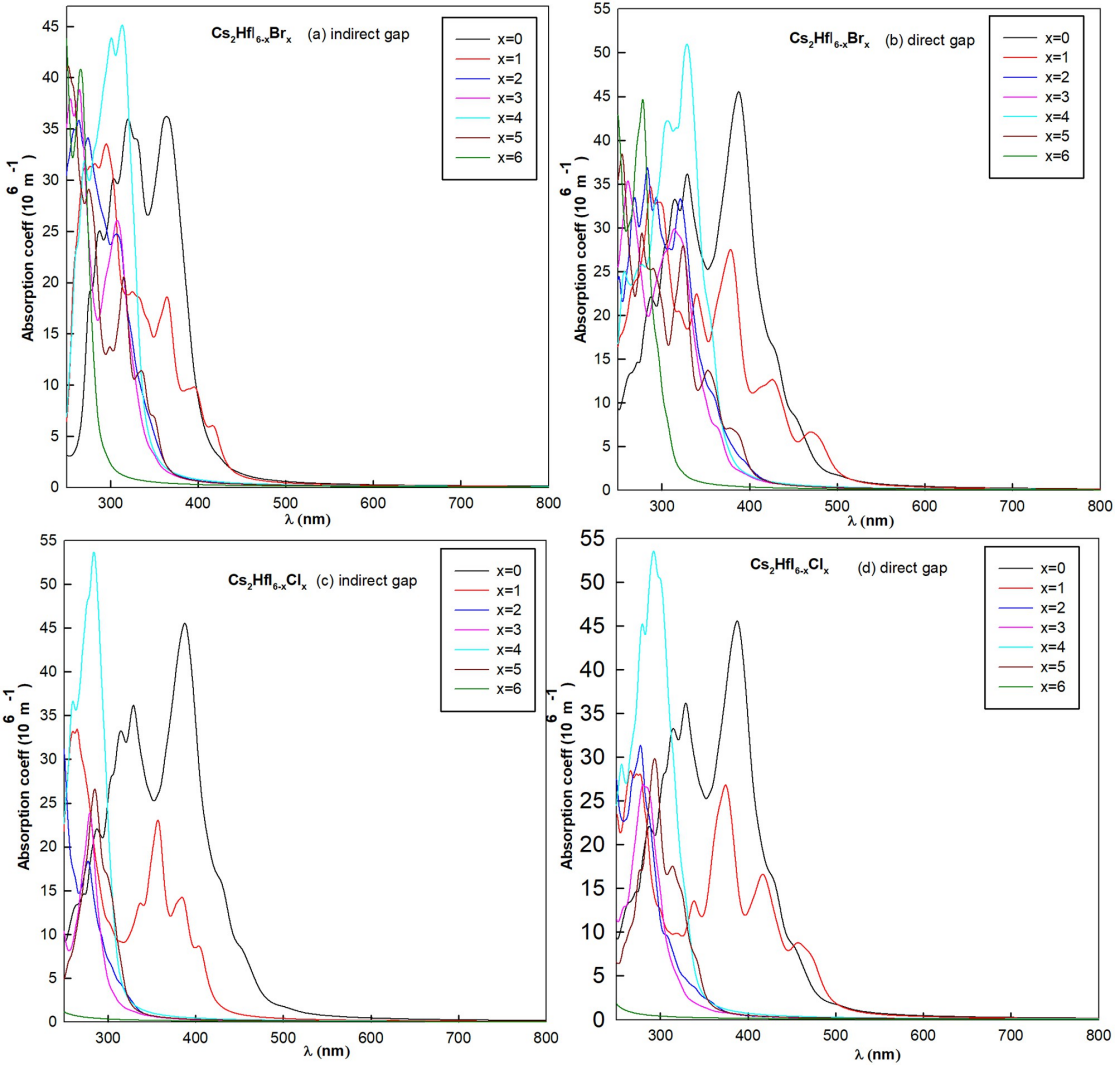
The optical spectrums of Cs_2_HfI_6−*x*_Br_*x*_ with (a) P = 0 and (b) phase transition pressure. (c) and (d) are the same conditions of Cs_2_HfI_6−*x*_Cl_*x*_.

**Fig 8:**
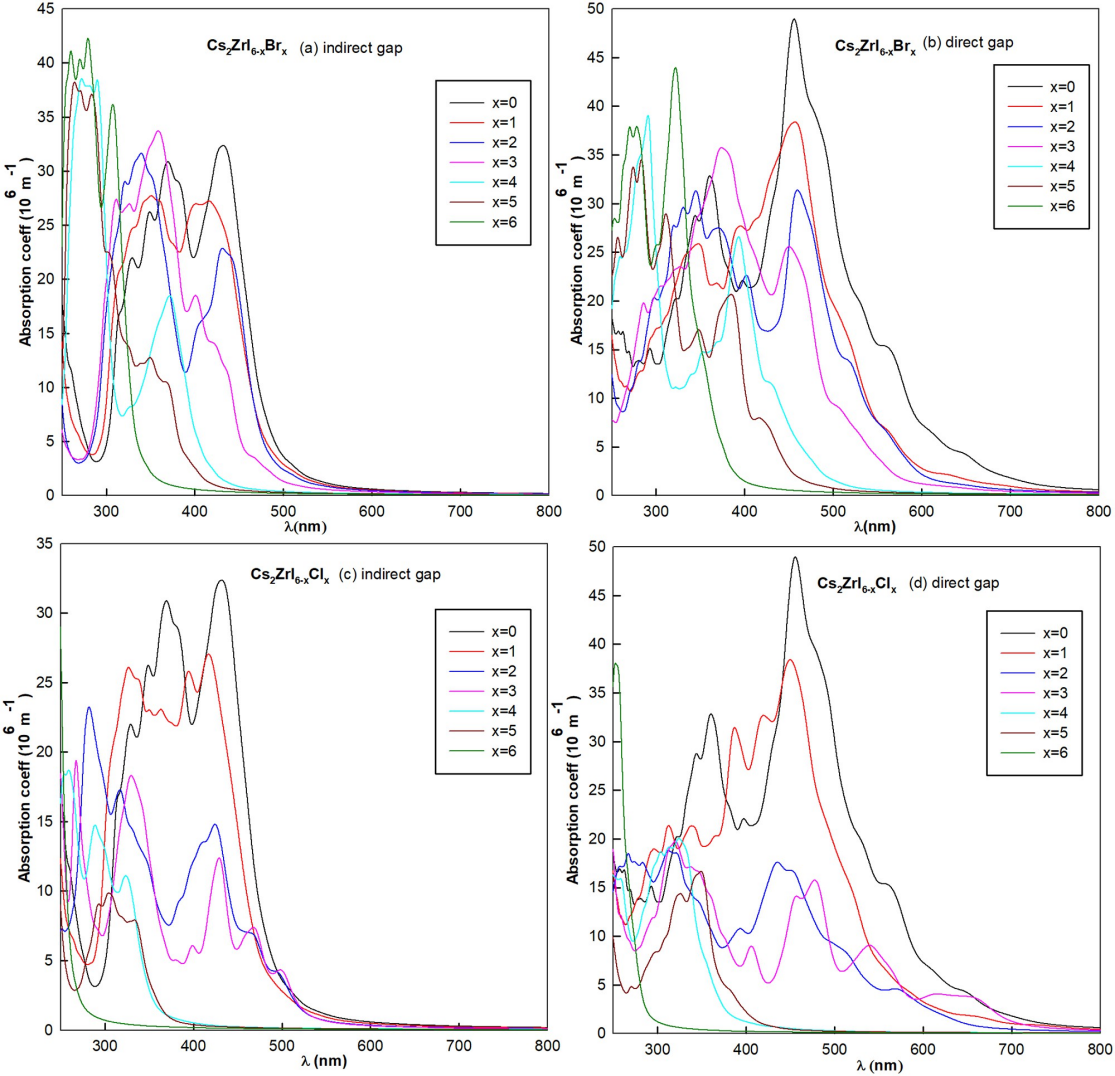
The optical spectrums of Cs_2_ZrI_6−*x*_Br_*x*_ with (a) P = 0 and (b) phase transition pressure. (c) and (d) are the same conditions of Cs_2_ZrI_6−*x*_Cl_*x*_.

In this section, the optical properties will be discussed in detail, and the optical absorption coefficients *I*(*ω*) defined through the linear response theory is calculated by the following equation:

$$\begin{array}{c} I\left(\omega \right)=\sqrt{2}\omega {\left[\sqrt{\epsilon_{1}^{2}\left(\omega \right)+\epsilon_{2}^{2}\left(\omega \right)}-\epsilon_{1}\left(\omega \right)\right]}^{1/2} \end{array}$$ (3)

where *ω* is the frequency of energy, *I*(*ω*) stands for the absorption coefficient, *ϵ*_1_(*ω*) and *ϵ*_2_(*ω*) are the real and imaginary parts of dielectric function, respectively. The absorption coefficients of 26 kinds halide perovskites with P = 0 and phase transition pressure are shown in Figs 7 and 8.

As can be seen from the spectrum diagram in Fig 7(a)–7(d), Cs_2_HfI_6−*x*_Br_*x*_ has better optical absorption for high frequency light with wavelength between 300–450*nm*. With the increase of Br concentration, absorption region are mainly move to short-wave area and peak value of absorption coefficient has been reduced at the same time. Along with the phase transition of indirect-direct band gap semiconductor, the peak value of absorption coefficient was increased. Overall, absorption coefficient of Cs_2_HfI_2_Br_4_ is the largest, but the main absorption area is ultraviolet spectrum within 320–360*nm*. If the halogen doping replaced by Cl atom, the absorption peak increased while the spectrum has obvious blue shift among all the absorption wavelength. Which means the absorption properties greatly reduced in the visible light region.

Fig 8(a)–8(d) is the absorption coefficient of Cs_2_ZrI_6−*x*_X_*x*_ compounds. Generally speaking, feature of spectral line has a similar trend with Cs_2_HfI_6−*x*_X_*x*_. But Cs_2_ZrI_6−*x*_X_*x*_ has better optical absorption property with the visible light absorption wavelength about 300–600*nm*. As to direct band gap materials, the absorption width increase to 700*nm* (Fig 8(b) and 8(d)). From Fig 8(a)–8(b), it is clear that all the materials have good light absorption properties, especially for direct band gap semiconductors. While the light absorption property of Cs_2_ZrI_6−*x*_Cl_*x*_ compounds is not well (Fig 8(c) and 8(d)). The absorption of visible light narrowed sharply with the increase of doping concentration, at the same time, peak value of absorption coefficient has decreased more than 40%. This may be caused by bigger radius of Cl- atom.

In general, Zr-based compounds Cs_2_ZrI_6−*x*_Br_*x*_ are more suitable for solar cells. Considering with the band characters of materials, we can conclude that Cs_2_ZrI_4_Br_2_ is an excellent candidate for optoelectronic applications.

## Conclusion

First principles calculation employing GGA-PBE method have been implemented to compute the electronic structures of 26 kinds of perovskites Cs_2_BI_6−*x*_X_*x*_, considering B = Hf and Zr cations, X as Cl and Br anion. The band gap can be regulated obviously by these ions. And all the materials suffer from indirect to direct band gap semiconductors under the impact of pressure. Calculated absorption coefficient trends identified that direct band gap materials of Cs_2_ZrI_6−*x*_Br_*x*_ have good optical absorption properties, especially Cs_2_ZrI_4_Br_2_ is a perfect candidate for perovskite solar cells. In conclusion, the trends in band gaps and absorption characters with the influence of pressure and halide ion studied in the paper can be useful in guiding further work in halide perovskite solar cells.

## Supporting information

S1 File (EPS)

S2 File (EPS)

S3 File (ZIP)

S4 File (ZIP)
